# Phospholipase D2 Modulates the Secretory Pathway in RBL-2H3 Mast Cells

**DOI:** 10.1371/journal.pone.0139888

**Published:** 2015-10-22

**Authors:** Claudia Maria Meirelles Marchini-Alves, Valeria Cintra Barbosa Lorenzi, Elaine Zayas Marcelino da Silva, Vivian Marino Mazucato, Maria Celia Jamur, Constance Oliver

**Affiliations:** Department of Cell and Molecular Biology and Pathogenic Bioagents, Ribeirão Preto Medical School, University of São Paulo, Ribeirão Preto, São Paulo, Brazil; Universidad del Pais Vasco, SPAIN

## Abstract

Phospholipase D (PLD) hydrolyses phosphatidylcholine to produce phosphatidic acid (PA) and choline. It has two isoforms, PLD1 and PLD2, which are differentially expressed depending on the cell type. In mast cells it plays an important role in signal transduction. The aim of the present study was to clarify the role of PLD2 in the secretory pathway. RBL-2H3 cells, a mast cell line, transfected to overexpress catalytically active (PLD2CA) and inactive (PLD2CI) forms of PLD2 were used. Previous observations showed that the Golgi complex was well organized in CA cells, but was disorganized and dispersed in CI cells. Furthermore, in CI cells, the microtubule organizing center was difficult to identify and the microtubules were disorganized. These previous observations demonstrated that PLD2 is important for maintaining the morphology and organization of the Golgi complex. To further understand the role of PLD2 in secretory and vesicular trafficking, the role of PLD2 in the secretory process was investigated. Incorporation of sialic acid was used to follow the synthesis and transport of glycoconjugates in the cell lines. The modified sialic acid was subsequently detected by labeling with a fluorophore or biotin to visualize the localization of the molecule after a pulse-chase for various times. Glycoconjugate trafficking was slower in the CI cells and labeled glycans took longer to reach the plasma membrane. Furthermore, in CI cells sialic acid glycans remained at the plasma membrane for longer periods of time compared to RBL-2H3 cells. These results suggest that PLD2 activity plays an important role in regulating glycoconjugate trafficking in mast cells.

## Introduction

PLD has been implicated in different cellular functions that can be attributed either to its catalytic activity or direct interaction with other proteins [1, 2]. PLD’s enzymatic activity hydrolyzes phosphatidylcholine that results in phosphatidic acid. In mammals there are two isoforms, PLD1 and PLD2 which have a 50% homology, but play distinct roles depending on the cell type [3–8]. Blockage of PLD activity with a primary alcohol results in the arrest of vesicle transport from the ER to the Golgi complex, vesicle formation at the TGN (trans-Golgi network) and a reversible fragmentation of the Golgi complex [9–12]. Previous studies have shown that PLD2 is associated with the Golgi complex and by electronic microscopy PLD2 was localized at the rims of the Golgi complex in pituitary GH3 cells [13, 14]. PLD2 was also shown to regulate constitutive secretion in epithelial cells [15]. Previous work from our laboratory further demonstrated that PLD1 and PLD2 tightly regulate the morphology of the Golgi complex in duct cells from the parotid gland [16]. Also, PLD2 is essential for maintaining the morphology of the Golgi complex in rat RBL-2H3 mast cells [17]. In an effort to understand the role of PLD2 during mast cell activation, RBL-2H3 rat mast cells were used to overexpress PLD2 in the catalytic active and inactive form. Measurement of PLD activity was accessed by quantitation of phosphatidic acid. Cells that overexpressed PLD2 in the active form produced twice the amount of PA as its counterparts [18]. Therefore it was of interest to investigate if PLD2 can modulate glycoprotein and glycolipid trafficking through the secretory system using the above mentioned cell lines. N-acetylmannosamine-azide (ManNAz), a modified sugar that can be labeled with a fluorophore or a biotin, was used to metabolically label glycoconjugates [19]. ManNAz is the metabolic precursor of sialic acid and is incorporated in N- and O- linked glycans at the TGN. The role of PLD2 on glycoconjugate synthesis and trafficking was examined. The results show that PLD2 is important for the regulation of ManNAz glycan trafficking through the secretory pathway in RBL-2H3 mast cells.

## Materials and Methods

### Cells

RBL-2H3 cells, a rat mast cell line [20], as well as RBL-2H3 cells transfected to overexpress catalytically active PLD2 (PLD2CA; clone D2-WT-1) or catalytically inactive PLD2 (PLD2CI; clone D2/K758R-1), were generously provided by Reuben P. Siraganian, MD, PhD (National Institute of Dental and Craniofacial Research, National Institutes of Health, Bethesda, MD). Cells were grown as monolayers at 37°C in Dulbecco’s modified Eagle’s medium (DMEM) supplemented with 15% fetal calf serum, 0.434 mg/ml glutamine, and an antibiotic-antimycotic mixture containing 100 units/ml penicillin, 100 μg/ml streptomycin, and 0.25 μg/ml amphotericin B (all from Life Technologies, Gibco, Carlsbad, CA) in an humidified incubator with 5% CO_2_ in air. Transfected cells were selected with geneticin (0.4 mg/ml) (Sigma-Aldrich; St. Louis, MO).

### Antibodies, Fluorescent Markers, and Stains

The following primary antibodies were used: mouse mAb anti-GM-130 (4 μg/ml, Clone 35/GM130; BD Transduction Laboratories, San Jose, CA); mouse mAb anti-TGN38 (3 μg/ml, detects specific protein present in the trans Golgi network; Clone 2/TGN38; BD Transduction Laboratories); mouse mAb AA4 (2.5μg/ml; BD Biosciences, San Jose, CA) which recognizes mast cell specific gangliosides; and mAb AD1, which recognizes CD63 (5 μg/ml; BD Biosciences); Goat- IgG-HRP (1:20,000) used for Western blots was purchased from Jackson ImmunoResearch Laboratories (Port Washington, PA): Goat anti-mouse IgG F(ab′)2–Alexa 594 (1:300 in PBS; Life Technologies, Molecular Probes) was used for immunofluorescence.

### Immunofluorescence

3.5 × 10^4^ cells were plated on 13 mm glass coverslips, and cultured for 16 h. Cells were rinsed in PBS, fixed for 20 min with 2% formaldehyde (EM Sciences, Hatfield, PA) in PBS, rinsed again, and permeabilized with 0.01% saponin (Sigma-Aldrich) in PBS for 20 min. Next, cells were rinsed twice in PBS and incubated for 30 min at room temperature in PBS containing 1% BSA (Sigma-Aldrich) and 5 μg/ml donkey-IgG (Jackson ImmunoResearch). Cells were then labeled with primary antibodies diluted in PBS containing 1% BSA for 1 h at room temperature. They were rinsed in PBS, followed by incubation for 45 min at room temperature with the secondary antibodies diluted in PBS. Cells were then rinsed in PBS and coverslips mounted with Fluoromount-G (EM Sciences). Samples were analyzed using a Leica SP2 or SP5 scanning confocal microscope (Leica Microsystems GmbH, Wetzlar Germany). Samples incubated without primary antibody served as controls. All controls were negative. The samples immunostained with anti-AA4, CD63, GM130 and TGN38 were examined with Olympus BX50 microscope (Olympus Corporation of the Americas, Center Valley, PA) equipped with a Nikon DXM 1200 digital camera (Nikon USA, Melville, NY). In S1D Fig, the cell outline was drawn with the freehand tool in Photoshop CS6 (Adobe Systems Incorporated, San Jose, CA).

### siRNA Knockdown

The PLD2 siRNA used was ON-TARGET plus SMART pool L-089287-00-0010 (Dharmacon—Thermo Fisher Scientific, Lafayette, CO). 0.8 x 10^5^ RBL-2H3 cells/well were plated in 6 well plates for 16 h. Transfections were performed using Lipofectamine RNAiMAX (Life Technologies) with 20nM PLD2 siRNA. After 3 days RNA was extracted to verify the PLD2 knockdown level. As a negative control siRNA ON-TARGET plus Non-targeting (Dharmacon) was used.

### Detection of PLD2 mRNAs by Real-Time PCR

Total RNA was purified from each cell line using the RNeasy Mini kit (Qiagen; Valencia, CA) according to the manufacturer’s instructions. For cDNA synthesis and mRNA analysis, 20 ng of RNA was subjected to real-time RT-PCR using QuantiFast SYBR Green RT-PCR (Qiagen). For all RT-PCR analysis, actin mRNA was used to normalize RNA inputs. Primer sequences are as follows:

rat PLD2 forward (5′-ATGACTGTAACCCAGACGGACCTC-3′);

rat PLD2 reverse (5′-CAGCTCCTGAAAGTGTCGGAATTT-3′);

rat actin forward (5′- ACAGGATGCAGAAGGAGATTAC-3′);

rat actin reverse (5′- ACAGTGAGGCCAGGATAGA-3′).

### Image Analysis

The fluorescence intensity was quantified using Image-Pro Plus, ver. 7.0 (Media Cybernetics; Silver Spring, MD). A region of interest was selected and the fluorescence intensity measured. A minimum of 50 cells was analyzed for each experimental condition. For quantification of colocalization Manders’ Colocalization coefficients M1/M2 were calculated using ImageJ (National Institutes of Health, http://rsb.info.nih.gov/ij/). M1 is the percentage of above-background pixels in the green channel that overlap above-background pixels in the red channel. ManNAz was considered the green channel and the organelle markers were considered the red channel. The organelle markers were CD63 for secretory granules, GM130 for cis Golgi, TGN38 for the trans Golgi network, and AA4 for GD1b derived gangliosides. A minimum of 15 cells was analyzed for each colocalization assay.

### Metabolic incorporation of ManNAz and GlcNAz into glycans

Intracellular glycoproteins were metabolically labeled using the Click-IT® Metabolic Labeling System (Life Technologies). This procedure consists of two parts: 1) the modified sugar that contains an azide chemical group which is added to the culture media and is incorporated by cells during glycoprotein synthesis; 2) the modified sugar is detected with a probe containing an alkyne chemical group attached to a fluorophore or biotin [21, 22]. All the procedures where performed according to the manufacturer’s directions.

### Detection of modified sugars with Alexa 488-alkyne probe

3.5 × 10^4^ cells were plated on 13-mm round glass coverslips, and incubated with 50μM of ManNAz or GlcNAz in DMEM for up to 3 days. Cells were rinsed twice in PBS, fixed for 20 min with 2% formaldehyde (EM Sciences) in PBS, and rinsed again. For some experiments, cells were permeabilized with 0.01% saponin (Sigma-Aldrich) in PBS for 20 min to enable the intracellular localization of the modified sugars.

For the pulse-chase experiments, 3.5 × 10^4^ cells were plated on 13-mm round glass coverslips and pulse-labeled with 50μM of ManNAz or GlcNAz in DMEM for 1 h. Next, cells were rinsed twice in PBS, and fresh DMEM was added for the chase period of 1, 3, 12, 24, 36, 48, 60 and 72 h. Later, cells were rinsed twice in PBS, fixed for 20 min with 2% formaldehyde (EM Sciences) in PBS, and rinsed again. For some experiments cells were permeabilized with 0.01% saponin (Sigma-Aldrich) in PBS for 20 min for intracellular localization of the modified sugars. To detect the modified sugars, the Click-IT® Cell Reaction Buffer (Life Technologies) was used to conjugate ManNAz or GlcNAz with the Alexa-488 alkyne probe, according to the manufacturer’s instructions. 1μM of Alexa 488-alkyne (Life Technologies) was used to visualize the modified sugars by fluorescence microscopy. For colocalization experiments, the immunofluorescence protocol was used as described before. As a negative control, RBL-2H3 cells were not incubated with the modified sugars, but the conjugation with the probe was performed. There was no nonspecific labeling.

### Detection of modified sugars with Biotin-alkyne probe

Cells were plated in 100mm tissue culture dishes (Corning Life Sciences, Union City, CA) and incubated with 50μM of ManNAz or GlcNAz in DMEM for 3 days. Next cells were rinsed twice in PBS, and detached with 2mM EDTA in PBS for 15 min. Cells were washed twice in PBS and lysed with RIPA buffer (Sigma-Aldrich) for 1 h on ice. The lysates were centrifuged and the supernatant was transferred to a new 1.5ml vial. The amount of protein in the lysates was quantified by the Bradford method (BioRad, Hercules, CA). The modified sugars were detected by conjugation with a Biotin-alkyne probe using the Click-IT Protein Reaction Buffer (Life Technologies) according to manufacturer’s instructions. To detect the modified sugars, 40μM of the Biotin-alkyne probe was used. Lysates were solubilized in 750μL sample buffer (125mM Tris; 4% SDS; 10% glycerol; 0.006% Coomassie blue and 1.8% β-mercaptoethanol) for 5 min at 70°C. The proteins were separated electrophoretically on 7.5% or 15% gels and transferred to Hybond membranes (GE-Healthcare Life Sciences). After transfer, the membranes were blocked for 1 h at room temperature in TBS (0.05 M Tris-HCl, 0.15 M NaCl [pH 7.5],) containing 5% BSA and 0.05% Tween 20. After blocking, the membranes were incubated for 1 h at room temperature with streptavidin-HRP (Life Technologies) diluted in TBS at 1:25,000. Membranes were then washed 10 times in TBS and developed using chemiluminescence (ECL-GE Healthcare). Mean optical density of the blots was determined using Adobe Photoshop 7.0 (Adobe Systems, San Jose, CA).

### Statistical analysis

The data are reported as mean ± standard error and statistically analyzed by *t*-test or one-way ANOVA using Tukey’s test. A *p* < 0.05 was considered statistically significant.

## Results

### ManNAz is incorporated into glycoproteins in the biosynthetic secretory pathway

**Fig 1:**
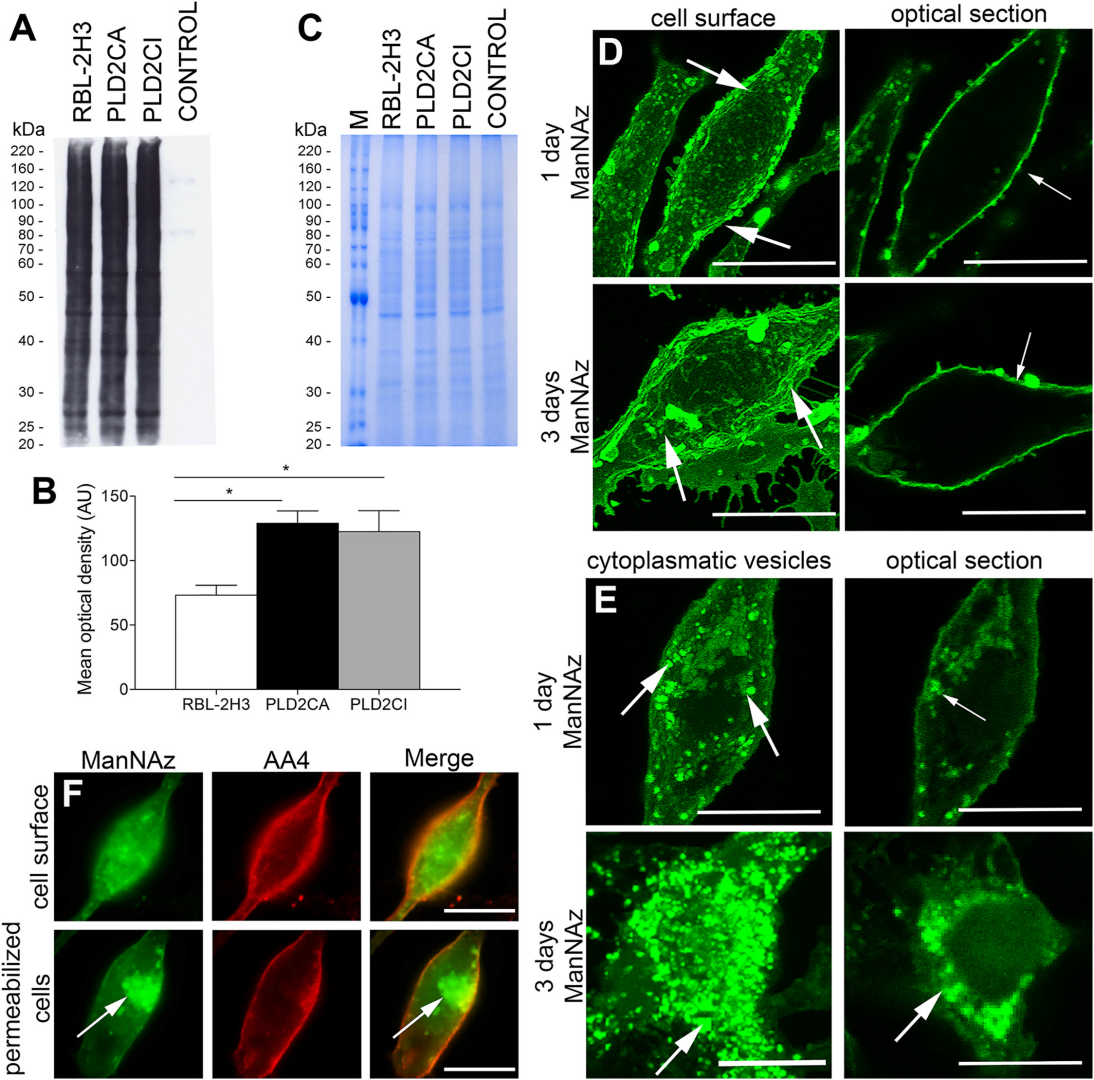
ManNAz is incorporated in the biosynthetic secretory pathway in RBL-2H3 mast cells. (A) After 3 days of ManNAz incubation, cells were lysed and blotted with streptavidin-HRP. In the control lane RBL-2H3 cell lysate was cultured in the absence of ManNAz. (B) ManNAz was incorporated in all cell lines, with a higher degree in PLD2CA and PLD2CI cells. P = 0.0148. (C) The cell lysates were applied to polyacrylamide gels and stained with Coomassie blue to verify equal protein loading. (D) RBL-2H3 cells were incubated with ManNAz for 1 or 3 days. ManNAz-Alexa 488 was localized at the plasma membrane (arrows). The right column shows one plane of the confocal image, to better visualize ManNAz-Alexa 488 on the membrane (Bars: 10μm). (E) RBL-2H3 cells were incubated with ManNAz for 1 or 3 days. To visualize ManNAz subcellular localization cells were permeabilized. ManNAz-Alexa 488 can be seen in a juxtanuclear region and inside cytoplasmic vesicles (arrows). The right column shows one plane of the confocal image, to better visualize ManNAz-Alexa 488 inside the cell (Bars: 10μm). (F) ManNAz-Alexa 488 colocalizes with GD1b derived gangliosides labeled with anti-mouse IgG conjugated to Alexa 594 on the plasma membrane (Bars: 10μm).

In order to determine if PLD2 plays a role in glycoprotein synthesis, the biosynthetic secretory pathway through the Golgi complex was investigated. Glycans were labeled with modified monosaccharide N-acetylmannosamine (ManNAz) or N-acetylglucosamine (GlcNAz) added to cell culture for uptake. ManNAz is the metabolic precursor of sialic acid and is incorporated in the final stages of glycosylation in the Golgi complex, more specifically, the TGN [19]. Although N-acetylglucosamine (GlcNAc) is an abundant component of N- and O-linked glycans, GlcNAz is not significantly incorporated into these glycans in mammalian cells [23] being preferentially added to proteins in the cytoplasm and nucleus at serine and threonine residues [24]. In order to confirm if the modified sugars were being incorporated into cell glycoproteins, PLD2CA, PLD2CI and RBL-2H3 cells were incubated in presence of ManNAz or GlcNAz and glycoproteins were analyzed from whole cell lysates (Fig 1A–1C and S1A and S1B Fig). While both sugars were incorporated into glycoproteins, incorporation of GlcNAz was much lower than ManNAz in all cell lines. The incorporation of ManNAz was slightly higher in the PLD2CA and PLD2CI cells than in the RBL-2H3 cells (Fig 1B). The subcellular localization of ManNAz was also determined in RBL-2H3 cells by confocal microscopy. In non-permeabilized RBL-2H3 cells ManNAz was present exclusively on the cell surface (Fig 1D). The localization of ManNAz was confirmed in a single plane in the z projection of the confocal image series (Fig 1D). Permeabilized RBL-2H3 cells showed ManNAz glycans present in cytoplasmic vesicles, in the juxtanuclear region and in the plasma membrane (Fig 1E). ManNAz had the same levels of intensity after 1 or 3 days of incubation. When GlcNAz was visualized by confocal microscopy it was not present on the cell surface of non-permeabilized cells (S1C Fig). In permeabilized cells, GlcNAz was mainly dispersed throughout the cytoplasm. After 3 days of incubation with GlcNAz, the intensity inside the cells was much lower than after 1 day of incubation, indicating that the rate of degradation GlcNAz glycoconjugates may be faster than ManNAz. To further confirm that ManNAz was incorporated in the glycosylation pathway, RBL-2H3 cells were incubated for 3 days with ManNAz and then cells were immunolabeled with mAb AA4 [25], which binds GD1b-derived gangliosides present on the plasma membrane of RBL-2H3 cells. There was colocalization between the ManNAz and GD1b-derived gangliosides at the plasma membrane in both conditions (Fig 1F). However, there was no colocalization between GlcNAz and the GD1b-derived ganglioside on the plasma membrane, further indicating that GlcNAz was not incorporated in the ER-Golgi complex glycosylation pathway (S1D Fig). These results confirm that ManNAz was incorporated into plasma membrane glycans in the ER-Golgi complex biosynthetic pathway in RBL-2H3 cells.

**Fig 2:**
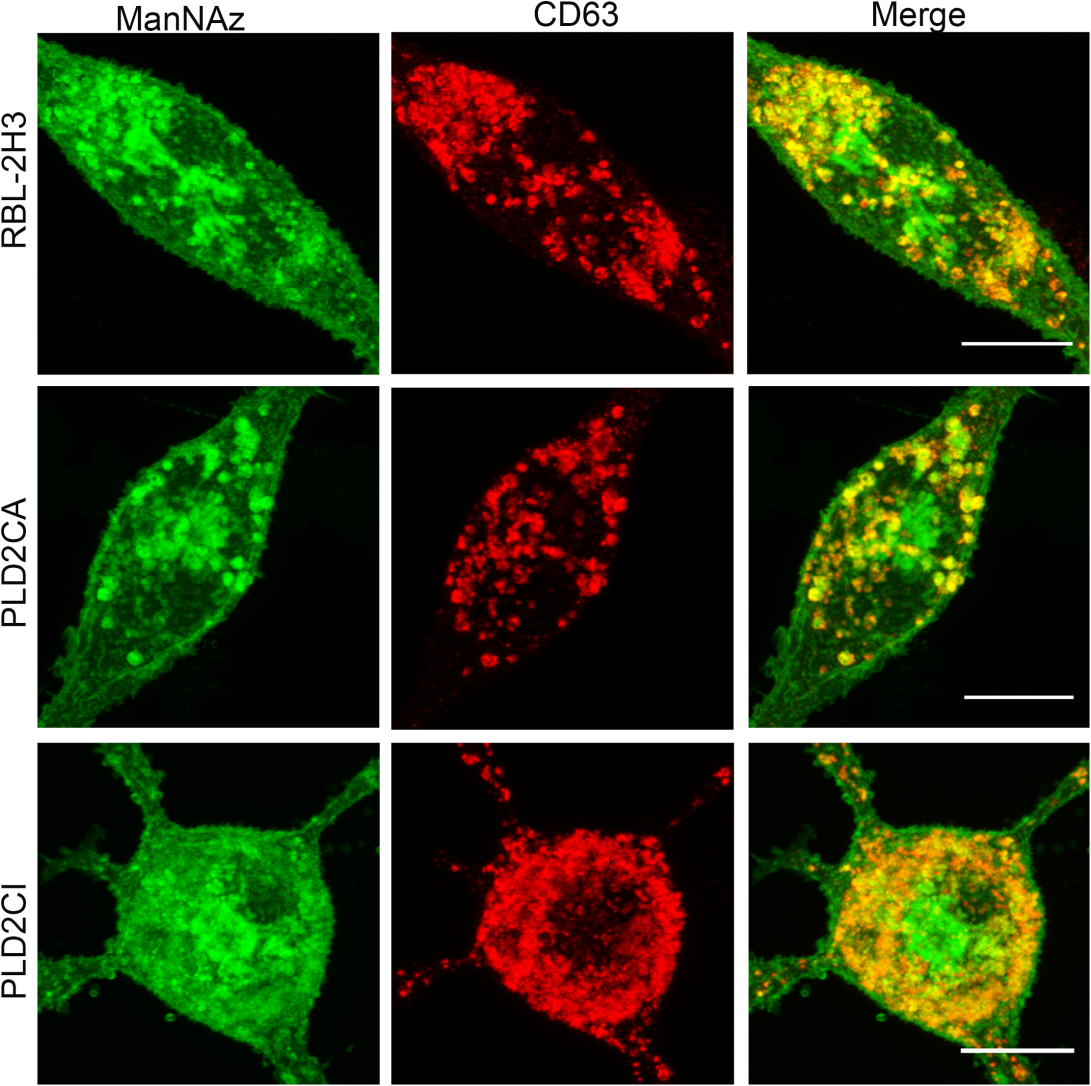
Identification of ManNAz in the juxtanuclear region of the cell and in secretory granules. Cells were labeled for 24 h with ManNAz and afterwards immunolabeled with anti-CD63. For confocal microscopy, ManNAz was coupled to Alexa 488 and anti-CD63 was detected with secondary antibody conjugated to Alexa 594. (Bars: 10μm).

**Fig 3:**
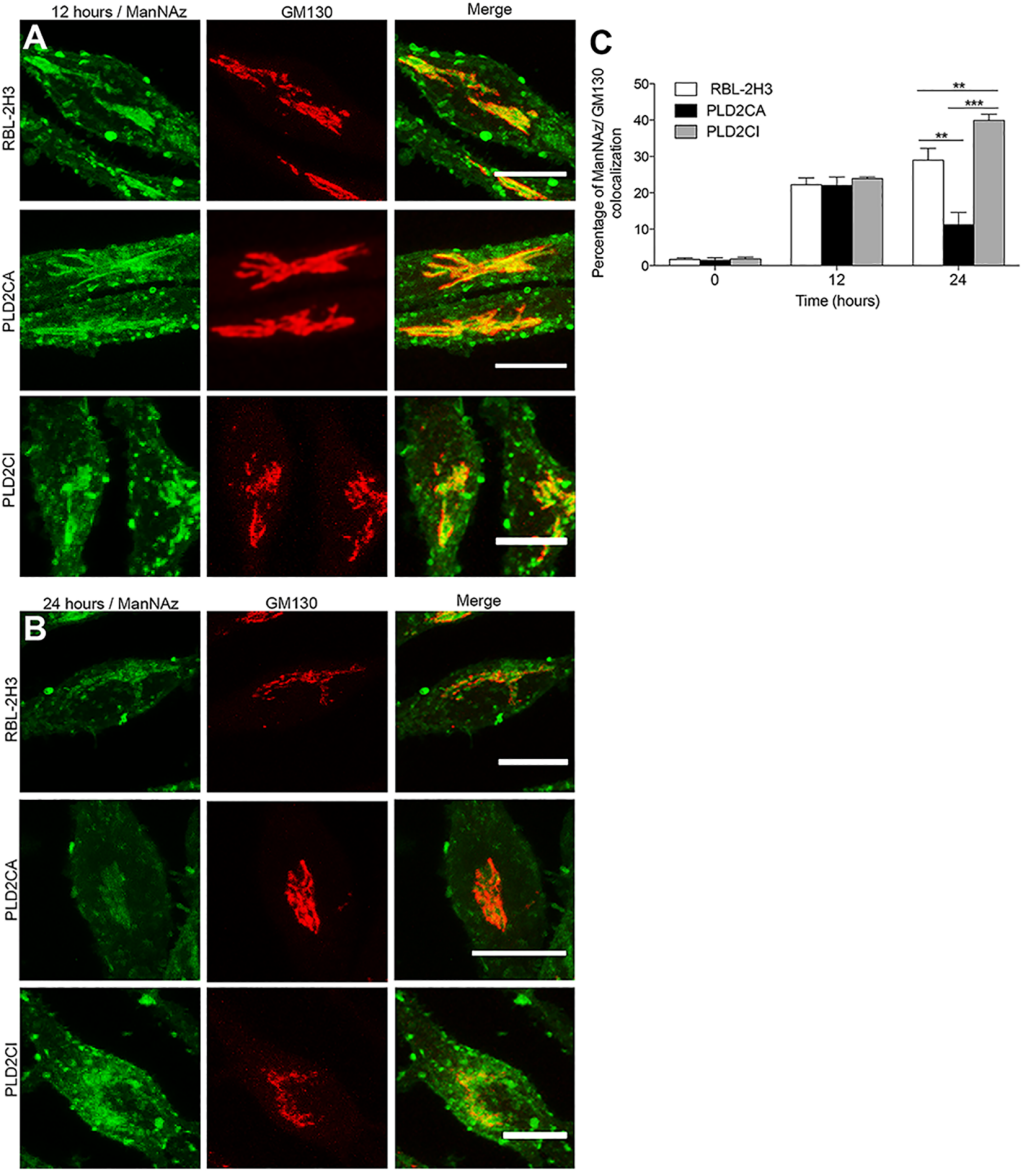
Characterization of ManNAz kinetics through the biosynthetic pathway. (A and B) In order to confirm that ManNAz was being incorporated, cells were pulse-labeled for 1 h with ManNAz and chased for 12 and 24 h. The cells were then immunolabeled with anti-GM130. (Bars: 10μm). (C) The kinetics of the colocalization of ManNAz with GM130 shows that in PLD2CA cells ManNAz quickly leaves the cis-Golgi, whereas in the PLD2CI cells ManNAz remains longer in the cis-Golgi. Values for Manders’ Colocalization coefficient M1 are shown. P = 0.0009. For confocal microscopy, ManNAz was coupled to Alexa 488 and anti-CD63, anti-GM130 was detected with secondary antibody conjugated to Alexa 594.

To better understand the role of PLD2 in mast cell glycosylation, stable cell lines derived from RBL-2H3 cells, overexpressing catalytically active or inactive PLD2 were used [17, 18]. The incorporation of ManNAz into organelles in the secretory pathway of these cell lines was confirmed. Cells were incubated with the modified sugar for 24 hours and then immunostained with antibody against CD63. ManNAz-Alexa 488 colocalized with CD63 in lysosomes and secretory granules (Fig 2), and there was no significant difference in the percentage of colocalization between the different cell lines (data not shown). Because ManNAz localized to a juxtanuclear region in RBL-2H3 cells, it was of interest to confirm if this region corresponded to the Golgi complex and TGN. The cells were incubated for 1 h in the presence of ManNAz and chased for an additional 12 h or 24 h (Fig 3A and 3B and S2A Fig). The cis region of the Golgi complex was then immunolabeled with anti-GM130, and the trans Golgi network was immunolabeled with anti-TGN38. ManNAz-Alexa 488 colocalized with both regions of the Golgi complex in all cell lines. These results taken together confirm that the ManNAz is incorporated into the secretory pathway in these cell lines.

### PLD2 modulates the transit of ManNAz glycoconjugates through the secretory pathway

To examine the trafficking of ManNAz through the secretory pathway, a pulse-chase experiment was performed. The different cell lines were incubated with ManNAz for 1 h and then chased for 12 or 24 h in culture media without the modified sugar (Fig 3A and 3B). The quantification of colocalization between ManNAz and the cis-Golgi marker indicated that around 20% of ManNAz colocalized with GM130 in all cell lines after 12 h of chase (Fig 3C). After 24 hours of chase there was an increase of 8% in the colocalization in RBL-2H3 cells, suggesting that ManNAz was accumulating in the cis-Golgi. However, in PLD2CA cells, the percentage of colocalization decreased to 13%, which is statistically significant in comparison to RBL-2H3 and PLD2CI cells. Thus indicating that in PLD2CA cells, glycans containing ManNAz leave the cis-Golgi region more rapidly, moving to other compartments or to the plasma membrane, when compared with RBL-2H3 cells. In PLD2CI cells there was an increase of 20% in the colocalization of ManNAz in the cis-Golgi after 24 h of chase. This was significantly higher than in RBL-2H3 cells. Indicating that ManNAz accumulated or was retained for longer periods in the cis-Golgi region when compared to RBL-2H3 cells. In the TGN region, the percentage of colocalization between ManNAz and TGN38 was around 30% in RBL-2H3 and PLD2CI cells (S2B Fig). Whereas in PLD2CA cells the colocalization was around 25%, indicating that ManNAz leaves the TGN38 region faster than in RBL-2H3 and PLD2CI cells. These results show that the catalytic activity of PLD2 is important for the regulation of ManNAz trafficking through the biosynthetic secretory system.

### In PLD2CI cells ManNAz glycans remain for longer periods of time at the cell surface

**Fig 4:**
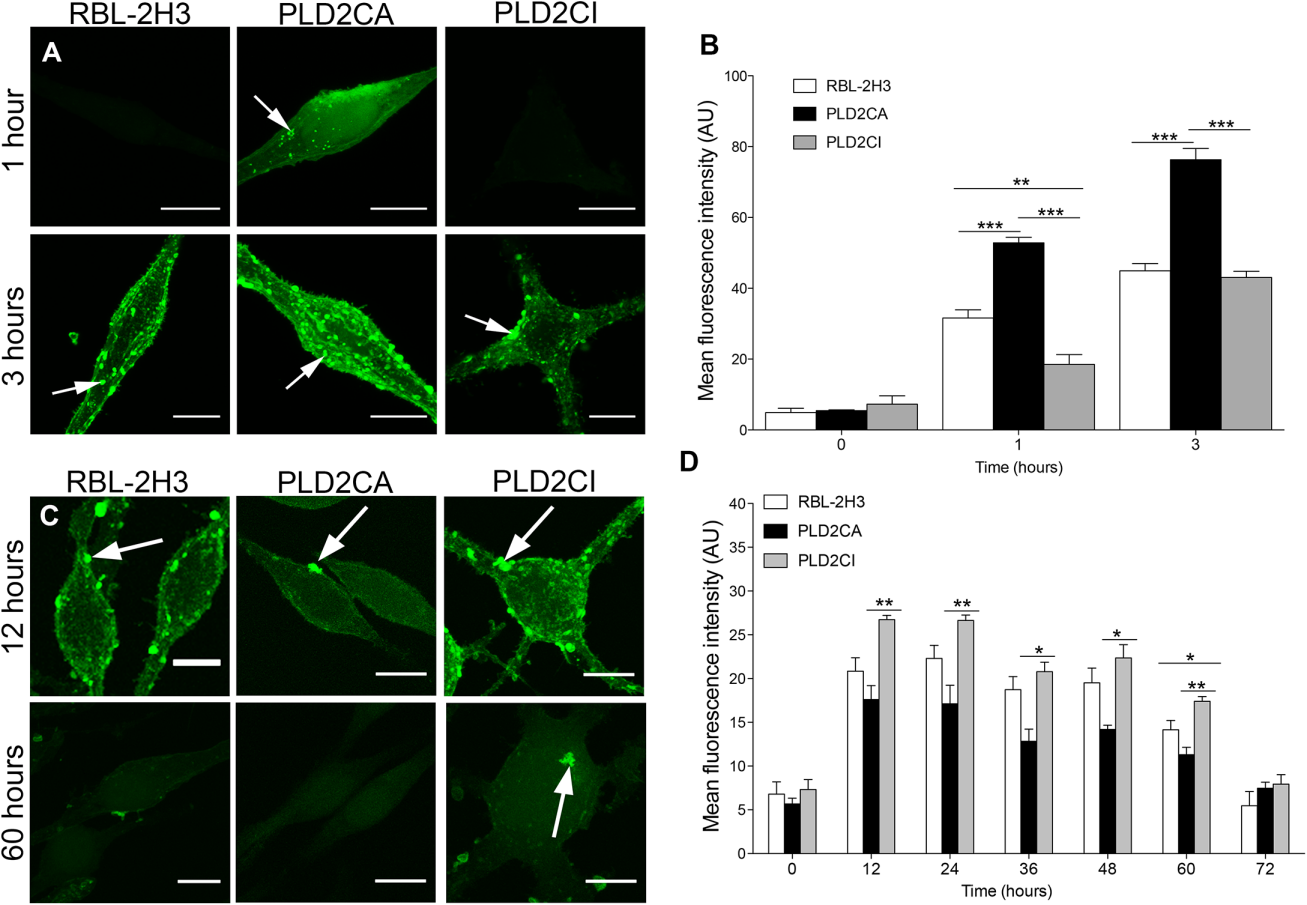
Trafficking of ManNAz towards the plasma membrane is regulated by PLD2. (A) Cells were pulse-labeled for 1 h with ManNAz, washed and chased for the indicated times. After 1 h of chase only PLD2CA cells had ManNAz labeling on the plasma membrane (arrow). With 3 h of chase all cell lines had ManNAz labeling on the plasma membrane (arrows) (Bars: 10μm). (B) Quantification of ManNAz at the cell surface. In PLD2CA cells, the amount of ManNAz on the plasma membrane was higher when compared to the other cells. P<0.0001 for 1 hour and 3 hours. (C) Cells were pulse-labeled for 1 h with ManNAz, washed and chased for the indicated times. ManNAz labeled the plasma membrane (arrows) (Bars: 10μm). (D) In PLD2CI cells there was more ManNAz on the cell surface throughout the investigated periods when compared with RBL-2H3 and PLD2CA. Moreover, in PLD2CA cells, there was a decreased amount of ManNAz on the cell surface in all the chase periods. P = 0.0122 for 12 hours. P = 0.0094 for 24 hours. P = 0.0337 for 36 hours. P = 0.0278 for 48 hours. P = 0.0020 for 60 hours. For confocal microscopy, ManNAz was coupled to Alexa 488.

It then became of interest to investigate the role of PLD2 in the trafficking of ManNAz glycans to the cell surface. The cells were pulsed with ManNAz for 1 h and chased for 1 and 3 h. In PLD2CA cells, after 1 h of chase, ManNAz was present on the cell surface. In contrast, ManNAz labeling on the cell surface of RBL-2H3 and PLD2CI cells was observed only after 3 h of chase (Fig 4A). However, the amount of ManNaz was still lower when compared to the PLD2CA cells. Also, after 3 h of chase, ManNAz aggregates were present on the cell surface of all cell lines. Therefore, PLD2 activity contributes to the delivery of glycoconjugates to the cell surface. These observations were confirmed by quantification of the fluorescence intensity of ManNAz (Fig 4B).

In order to have a better understanding of the ManNAz trafficking to the cell surface longer time periods of chase were also investigated. Cells were incubated with ManNAz for 1 h and chased with fresh medium without ManNaz and the cells fixed every 12 h for 72 h (Fig 4C and S2A Fig). After 12 h of chase ManNAz was present on the cell surface of RBL-2H3, PLD2CA and PLD2CI cells. Also, ManNAz appeared to aggregate on the cell surface. Furthermore, ManNAz labeling intensity was greater on the cell surface of RBL-2H3 and PLD2CI cells. After 60 h of chase ManNAz still was present on the cell surface of RBL-2H3 and PLD2CI cells. However, only small amounts of ManNAz were present on the surface of PLD2CA cells. Quantification of fluorescence intensity of the ManNAz showed significant differences between the cell lines (Fig 4D). After 12, 24 and 60 h of chase the intensity of ManNAz on the plasma membrane of PLD2CI was significantly higher when compared to RBL-2H3 cells. While in the PLD2CA cells, the intensity of ManNAz showed an overall decrease during the chase. These observations indicate that in PLD2CA cells, ManNAz may be delivered to the cell surface at earlier time points, or that glycans containing ManNAz are degraded more quickly when compared to the other cell lines. Therefore, PLD2 appears to contribute to the regulation of the delivery and maintenance of glycoconjugates to the cell surface.

### ManNAz remains longer in the biosynthetic secretory pathway in PLD2CI cells

**Fig 5:**
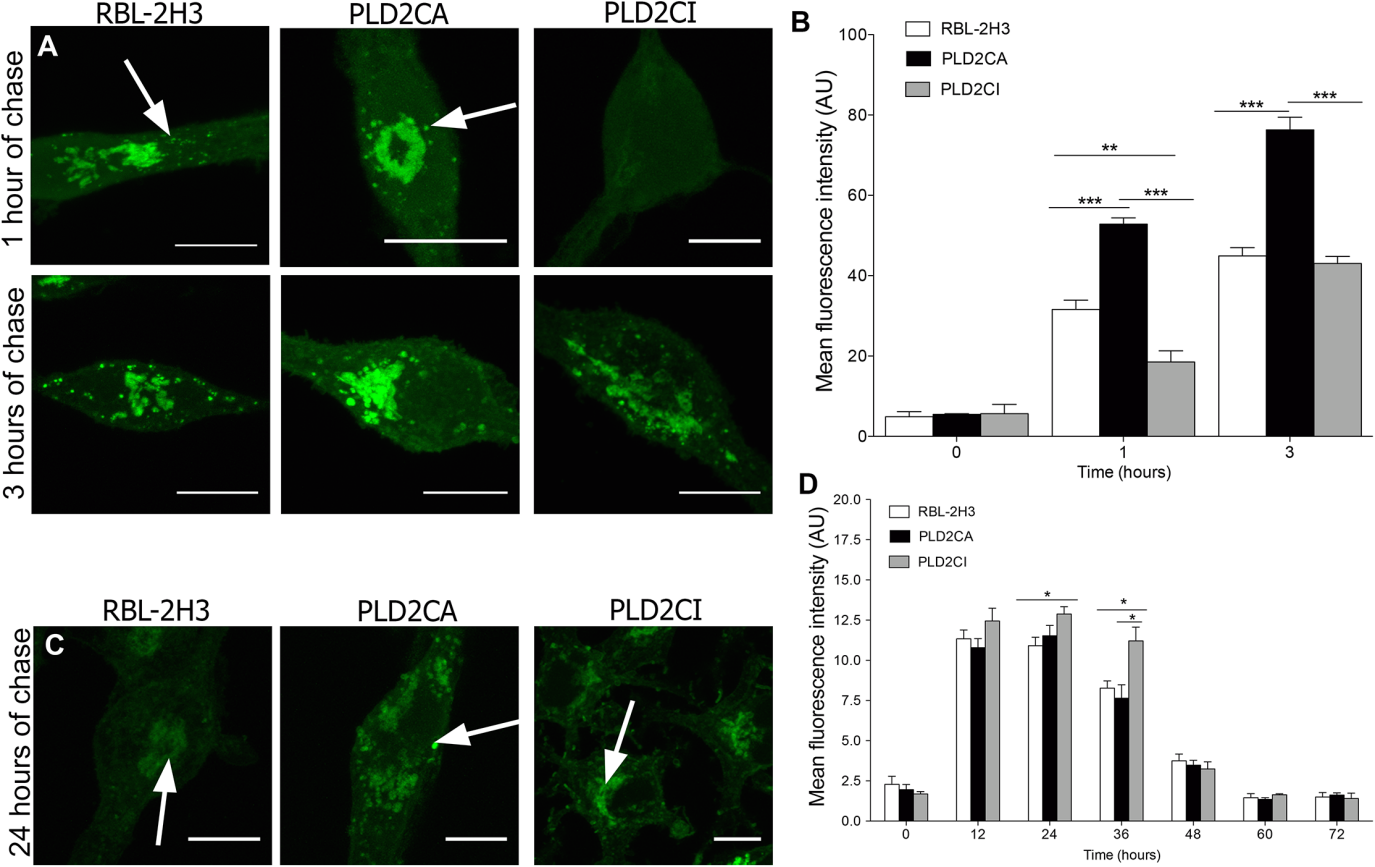
ManNAz trafficking is regulated by PLD2. (A) Cells were pulse-labeled for 1 h with ManNAz, washed and chased for 1 or 3 h. Cells were permeabilized to investigate ManNAz localization inside the cells. After 1 h of chase, ManNAz was localized juxtanuclearly in all cell lines. However, only PLD2CA and RBL-2H3 cells had cytoplasmatic vesicles containing ManNAz associated with the Golgi region (arrow). After 3 h of chase, all cell lines had ManNAz labeling in the Golgi complex as well as in cytoplasmatic vesicles in the same region (arrow). (Bars: 10μm). (B) In PLD2CA cells ManNAz concentration inside the cells was higher when compared to the other cells during the time periods analyzed. P<0.0001 for 1 and 3 hours. (C) Cells were pulse-labeled for 1 h with ManNAz, washed and chased for 24 h. Cells were permeabilized to investigate ManNAz localization inside the cells. ManNAz was localized juxtanuclearly and inside cytoplasmatic vesicles (arrows). (Bars: 10μm). (D) When the amount of ManNAz inside the cells was measured, there was more ManNAz labeling after 24 and 36 h of chase in PLD2CI cells than in RBL-2H3 and PLD2CA cells. P = 0.0463 for 24 hours. P = 0.0214 for 36 hours. For confocal microscopy, ManNAz was coupled to Alexa 488.

The previous results showed that the modified sugar remained longer at the plasma membrane of PLD2CI cells. Therefore, the intracellular dynamics of glycoconjugate trafficking was investigated (Fig 5). The intracellular localization of the ManNAz was first examined at 1 and 3 h of chase (Fig 5A). In RBL-2H3 and PLD2CA cells, after 1 h of chase ManNAz was observed in the juxtanuclear region and in vesicles located between this region and the plasma membrane. In these cell lines, after 3 h of chase, ManNAz can still be detected in the justanuclear region and in cytoplasmic vesicles. In contrast, in PLD2CI cells, although the modified sialic acid was observed in the juxtanuclear region at 1 h and 3 h of chase, vesicles associated with the juxtanuclear region were observed only after 3 h of chase. In all cell lines, the fluorescence intensity of ManNAz was increased after 1 h and 3 h of chase. However, the fluorescence intensity of the modified sugars was higher in PLD2CA cells than in PLD2CI and RBL-2H3 cells (Fig 5B). Therefore, in PLD2CA cells ManNAz is rapidly incorporated into glycans when compared to the other cell lines. In addition, less ManNAz was incorporated into the PLD2CI cells, ManNAz could de exiting the juxtanuclear region more slowly.

Longer periods of chase were also investigated. Cells were incubated with ManNAz for 1 h and chased with fresh medium without ManNAz for a total of 72 h (Fig 5C and S2B Fig). The amount of ManNAz present in the cells increased for the first 24 h of chase after which it began to decline in all cell lines. At 24 h of chase, ManNAz was localized in the juxtanuclear region as well as in cytoplasmic vesicles. In the remaining chase periods, the fluorescence intensity of ManNAz decreased in RBL-2H3, PLD2CA and PLD2CI cells (Fig 5D). However, in PLD2CI cells, the ManNAz intensity was significantly higher at 24 and 36 h of chase when compared to the RBL-2H3 cells. Thus, the traffic of glycans containing ManNAz is slower in PLD2CI and the exit from the Golgi region maybe slower than in the RBL-2H3 and PLD2CA cells. Therefore, catalytically active PLD2 appears to be required for trafficking of glycoconjugates through the biosynthetic pathway.

### PLD2 knockdown cells have the same phenotype as PLD2CI cells

**Fig 6:**
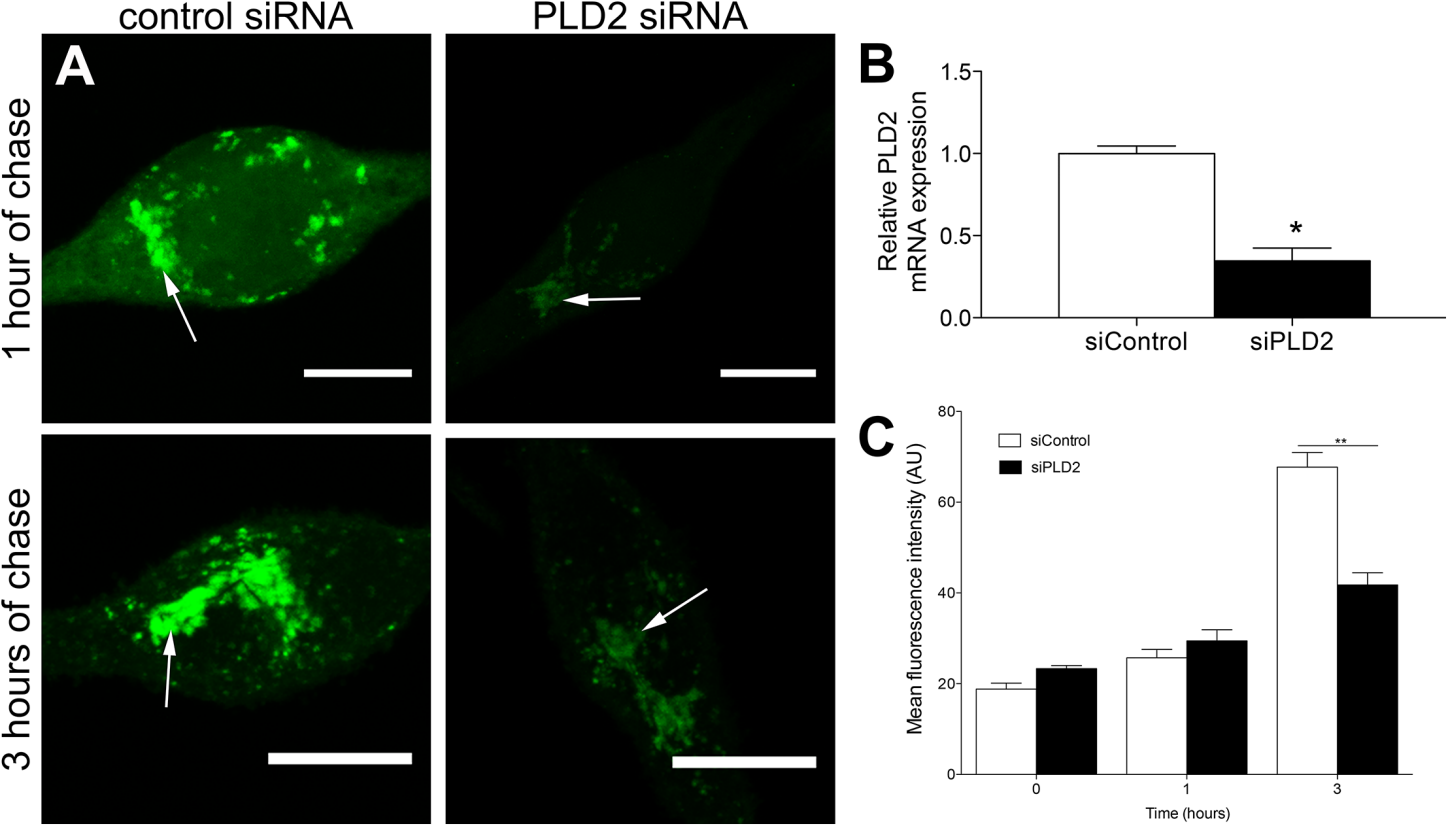
Knockdown of PLD2 in RBL-2H3 cells results in a phenotype similar to PLD2CI cells. (A) Cells were pulsed for 1 h with ManNAz and chased for 1 and 3 h (Bars: 10μm). (B) RBL-2H3 cells transfected with PLD2 siRNA had a 65% reduction in PLD2 expression when compared to control siRNA cells. P = 0.0279. (C) The fluorescence intensity of ManNAz in PLD2 siRNA cells was significantly lower than in control siRNA cells after 3 h of chase. P = 0.014 for 3 hours. For confocal microscopy, ManNAz was coupled to Alexa 488.

In order to confirm the participation of PLD2 in the trafficking of glycoproteins through the secretory biosynthetic pathway, PLD2 expression was silenced by RNA interference (Fig 6). Analysis by real-time RT-PCR showed a 65% reduction in PLD2 mRNA after transfection of RBL-2H3 cells with siRNA (Fig 6B). These cells were used to assess glycoprotein trafficking at 1 and 3 h of chase after ManNAz incorporation (Fig 6A and 6C). In RBL-2H3 cells treated with control siRNA, ManNAz was localized in the juxtanuclear region and in cytoplasmic vesicles after 1 h of chase. After 3 h of chase, although the localization remained the same, the fluorescence intensity of ManNAz increased in the juxtanuclear region and in the cytoplasmic vesicles. In the PLD2 knockdown RBL-2H3 cells, ManNAz was also located in the juxtanuclear region after 1 h, but there was no labeling of the cytoplasmic vesicles. Cytoplasmic vesicles near the juxtanuclear region were only observed after 3 h of chase in PLD2 knockdown RBL-2H3 cells. Furthermore after 3 h of chase the fluorescence intensity of ManNAz was significantly lower than in control RBL-2H3 cells. These results confirm that PLD2 activity is necessary for trafficking of glycoproteins through the secretory pathway.

## Discussion

The present study elucidates the participation of PLD2 in the transport of glycosylated materials to the cell membrane by overexpression of catalytically active and inactive forms of PLD2 as well as by knockdown of PLD2 in RBL-2H3 cells. This work shows that PLD2 activity, which results in phosphatidic acid production, is necessary for the correct trafficking of glycoconjugates to the plasma membrane. Reduced PLD2 activity results in the accumulation of glycoconjugates in the Golgi complex and retards their delivery to the plasma membrane. When PLD2 was knocked down, the ManNAz glycans remained for longer periods of time on the plasma membrane. PLD2 may modulate the half-life of these glycoconjugates on the plasma membrane by aiding their recycling back into the cell to be degraded or by facilitating their shedding into the extracellular medium [26].

Indeed in PLD2CA cells, which have previously been shown (to produce twice as much phosphatidic acid as its counterparts)[18], ManNAz traffic tends to be faster when compared to the control cells. This can be clearly seen in the pulse-chase experiments. After incubating cells with ManNAz for 1 hour for the pulse period, and adding new medium, waiting 24 hours for the chase period, it can be clearly seen that ManNAz no longer is in the cis Golgi. Most likely, because of the increased levels of PA, anterograde traffic of the ManNAz towards the cell surface is facilitated. Also, PLD2 knockdown decreased the amount of ManNAz present in the Golgi, indicating that there is less accumulation of ManNAz glycans in the Golgi. The evidence presented here provide a possible mechanistic explanation of how PLD2 activity regulates anterograde transport of proteins and lipids modified by ManNAz towards the plasma membrane.

The role of the Golgi complex in the secretory pathway has been extensively studied. The Golgi complex is the site where newly synthesized proteins and lipids, are processed and modified primarily by glycosylation [27, 28]. Cargo reaches the trans-Golgi network (TGN) were it is sorted for delivery to its final destination [28]. The TGN is also an intersection between the secretory and endocytic routes [29]. Cargo can be transported within the secretory system either by vesicles [30, 31] or tubular carriers [32].

As demonstrated in our previous work [17], cells that overexpress the catalytically inactive form of PLD2 have an altered Golgi morphology. The Golgi cisterns are dilated and are not parallel to each other. There are also fewer vesicles associated with the Golgi complex when compared to wild-type cells. PLD activity has been shown to be important for Golgi complex organization. The addition of primary alcohols to isolated Golgi membranes from rat liver cells resulted in the fragmentation of the Golgi membranes [11, 12, 33]. Primary alcohols such as butanol inhibit the production of phosphatidic acid by PLD. Other studies using permeabilized cells derived from growth hormone and prolactin-secreting pituitary GH3 cells [34], human promyelocytic leukemia cells [35], human neutrophils [36], mast cells [37], and regulatory T cells [38] have demonstrated that vesicle formation from the TGN during exocytosis was dependent on PLD. PLD2 activity has been shown to regulate the constitutive secretory pathway and vesicle transport between the trans cisternae and the apical region of the plasma membrane in human intestinal cells, HT29-c119A cells [15]. The overexpression of catalytically inactive PLD2 inhibited apical constitutive secretion by 70%. In rabbit mammary gland cells, treatment with butanol resulted in a decrease in casein secretion and serum acid protein [33]. Lipid modifying enzymes such as PLD are important in generating membrane curvature for vesicular trafficking. Diacylglycerol, lysophosphatidic acid and phosphatidic acid all affect membrane curvature, and recruit fission factors thereby altering membrane function [39–41].

PLD2 also has been directly linked to the formation of COPI coated vesicles from Golgi cisternae. In a COPI reconstitution system, using isolated Golgi membranes from PLD2 knockdown HeLa cells, the formation of COPI coated vesicles was impaired. Analysis of these membranes by TEM showed that the vesicle bud was forming, but the vesicles remained attached to the Golgi membrane [42, 43]. Thus suggesting that the production of phosphatidic acid by PLD2 is important for vesicle budding from the Golgi cisternae.

In the present study, the overexpression of catalytically inactive PLD2 delivered ManNAz slower to the plasma membrane and led to the accumulation of ManNAz glycans on the cell membrane where they remained for extended periods of time. The ManNAz glycans may remain on the cell surface because they are not undergoing endocytosis and being delivered to lysosomes for degradation. Indeed, it has been shown that PLD2 activity is involved in the endocytic process. Cells expressing catalytic inactive PLD2 have decreased internalization of epidermal growth factor receptor [44], mi-opioid receptor [45], mGluR1a metabolic glutamate receptor [46] and angiotensin II type 1 receptor [47].

Taken together the results of the present study indicate that PLD2 activity is important for the transport of glycoconjugates via the secretory pathway from the Golgi complex to the plasma membrane. Although inhibition of PLD2 activity did not interrupt the flow of glycoconjugates towards the membrane, it did decrease the flow. PLD2 has been linked to a plethora of cellular functions such as cell signaling, membrane remodeling and cancer. The current investigation provides additional evidence on the importance of PLD2 in cellular processes.

## Supporting Information

S1 Fig GlcNAz is incorporated in cytosolic and nuclear glycosylation in RBL-2H3 mast cells. (A) After 3 days of incubation with ManNAz or GlcNAz, cells were lysed and blotted with streptavidin-HRP. (B) ManNAz was incorporated in all cell lines, with a higher amount in PLD2CA and PLD2CI cells whereas, GlcNAz incorporation was lower. P = 0.0148 for ManNAz and P<0.0001 for GlcNAz. (C) RBL-2H3 cells were incubated with GlcNAz for 1 or 3 days. After 1 day of incubation with GlcNAz, the GlcNAz was localized in the cytoplasm with a punctate distribution in some regions (arrows). After 3 days there was a very low concentration of GlcNAz inside the cells (Bars: 10μm). (D) GlcNAz did not colocalize with GD1b derived gangliosides on the cell surface (Bars: 10μm). For blots ManNAz and GlcNAz were coupled to biotin. For confocal microscopy, GlcNAz was coupled to Alexa 488 and mAb AA4 was detected with donkey anti-mouse IgG conjugated to Alexa 594.
(TIF)

S2 Fig ManNAz glycoconjugates are localized at the trans Golgi network. (A) Cells were pulse-labeled for 1 h with ManNAz and chased for 12 h. Cells were then immunolabeled with anti-TGN38. For immunofluorescence microscopy, ManNAz was couples to Alexa 488 and anti-TGN38 was detected with secondary antibody conjugated to Alexa 594. (Bars: 10μm). (B) At 12 h of chase, there is a similar percentage of colocalization of ManNAz with TGN38 in all cells. P = 0.0398. Values for Manders’ Colocalization coefficient M1 are shown.
(TIF)

S3 Fig ManNAz pulse-chase. Cells were pulsed with ManNAz for 1 h and chased for 12, 24, 36 and 48 h. (A) Cells were not permeabilized to observe ManNAz at the cell surface (arrows). (Bars: 10μm). (B) Cells were permeabilized to observe the localization of ManNAz inside cells. For confocal microscopy ManNAz was coupled to Alexa 488. (Bars: 10μm).
(PSD)

S4 Fig Original uncropped blots and gel. Original film from Fig 1A was inverted to follow the same pattern as electrophoresis gel of same samples and facilitate visualization. Uncropped picture of gel from Fig 1C. Original film from S1A Fig.
(TIF)
