# Supplementary figures and images for: Phospholipase D2 Modulates the Secretory Pathway in RBL-2H3 Mast Cells
Source: PLoS One. 2015 Oct 22;10(10):e0139888. doi: 10.1371/journal.pone.0139888 (PMC4619593; doi:10.1371/journal.pone.0139888)

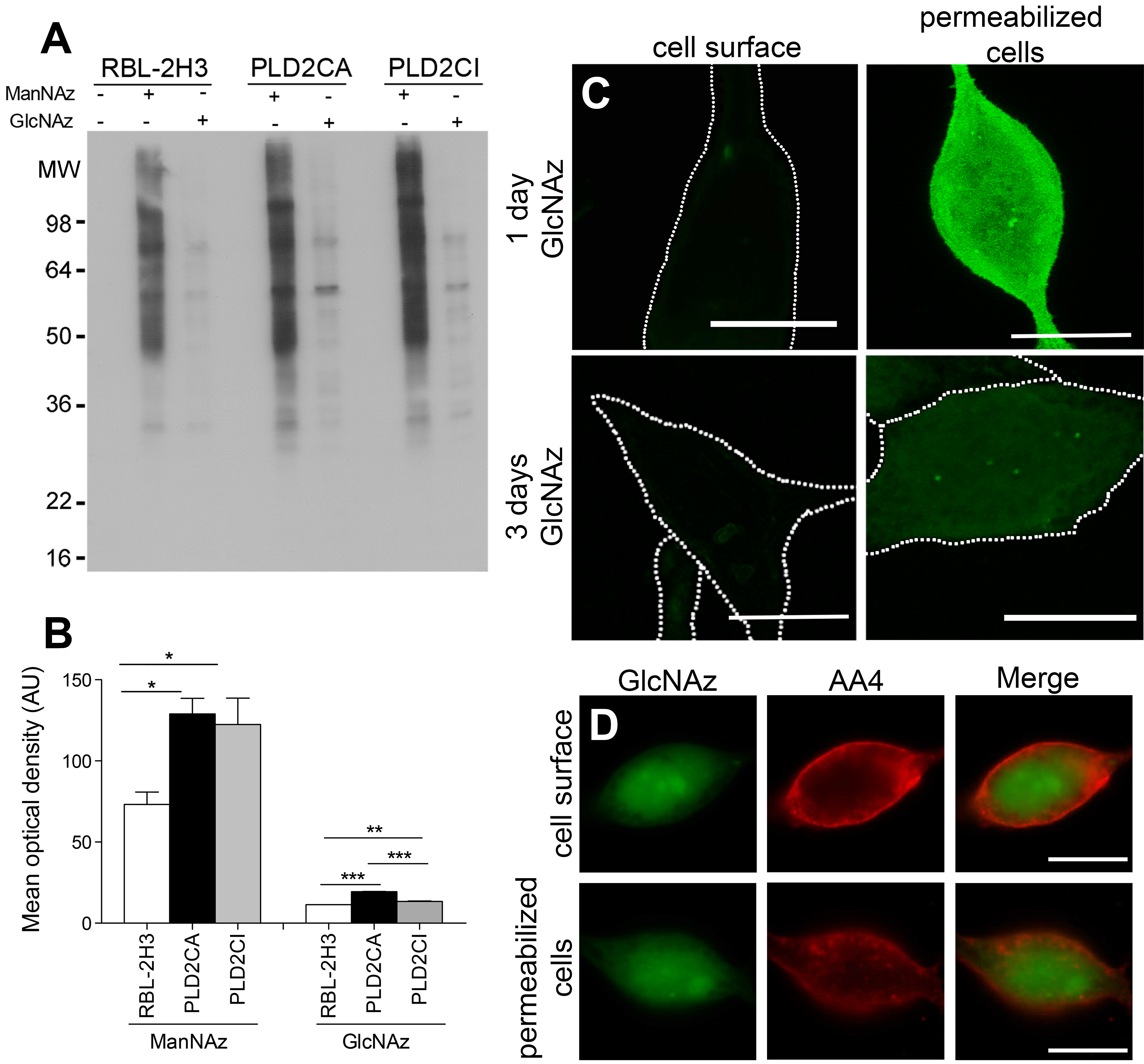

Supplement: S1 Fig — (A) After 3 days of incubation with ManNAz or GlcNAz, cells were lysed and blotted with streptavidin-HRP. (B) ManNAz was incorporated in all cell lines, with a higher amount in PLD2CA and PLD2CI cells whereas, GlcNAz incorporation was lower. P = 0.0148 for ManNAz and P<0.0001 for GlcNAz. (C) RBL-2H3 cells were incubated with GlcNAz for 1 or 3 days. After 1 day of incubation with GlcNAz, the GlcNAz was localized in the cytoplasm with a punctate distribution in some regions (arrows). After 3 days there was a very low concentration of GlcNAz inside the cells (Bars: 10μm). (D) GlcNAz did not colocalize with GD1b derived gangliosides on the cell surface (Bars: 10μm). For blots ManNAz and GlcNAz were coupled to biotin. For confocal microscopy, GlcNAz was coupled to Alexa 488 and mAb AA4 was detected with donkey anti-mouse IgG conjugated to Alexa 594. (TIF) [file pone.0139888.s001.tif]

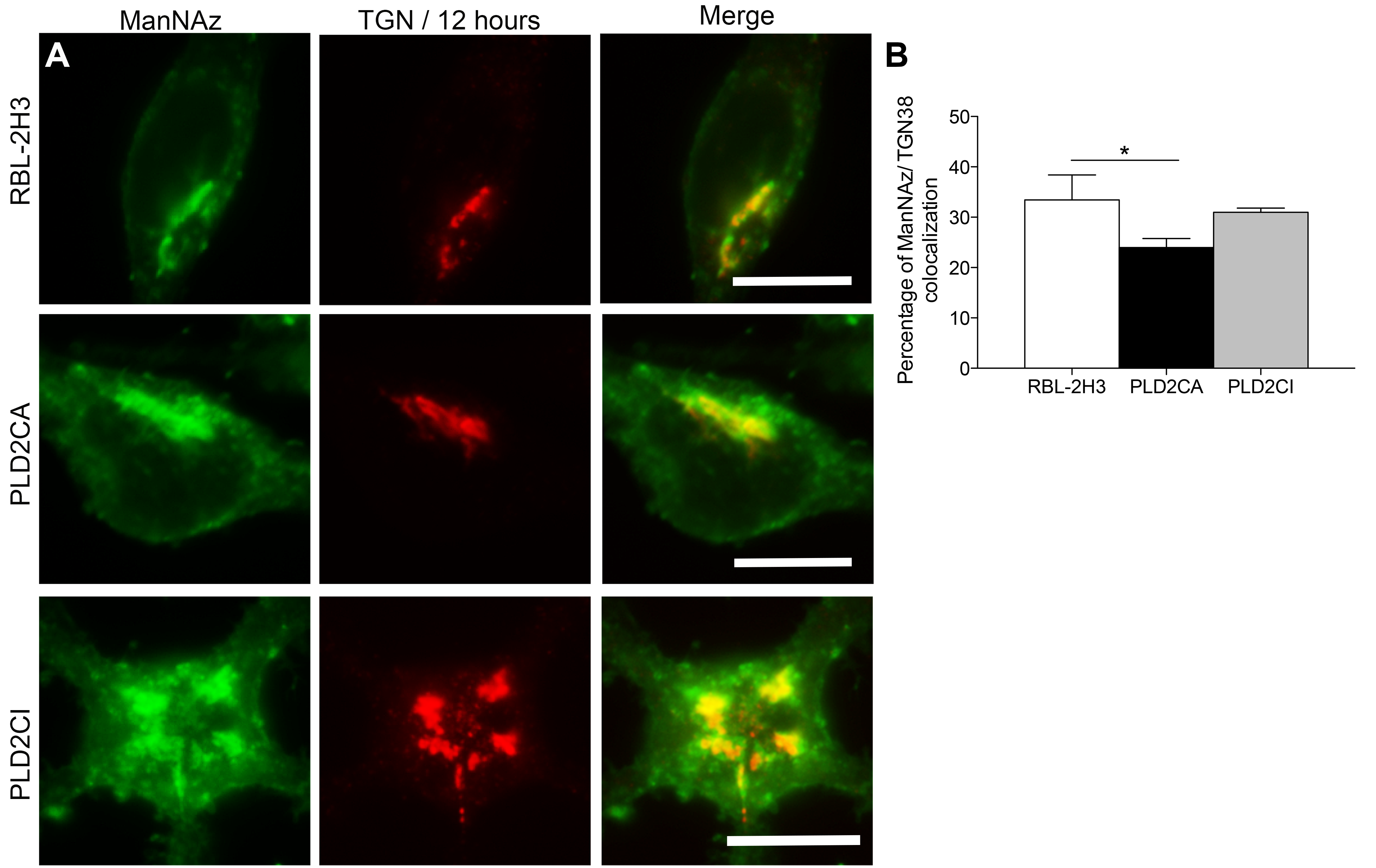

Supplement: S2 Fig — (A) Cells were pulse-labeled for 1 h with ManNAz and chased for 12 h. Cells were then immunolabeled with anti-TGN38. For immunofluorescence microscopy, ManNAz was couples to Alexa 488 and anti-TGN38 was detected with secondary antibody conjugated to Alexa 594. (Bars: 10μm). (B) At 12 h of chase, there is a similar percentage of colocalization of ManNAz with TGN38 in all cells. P = 0.0398. Values for Manders’ Colocalization coefficient M1 are shown. (TIF) [file pone.0139888.s002.tif]

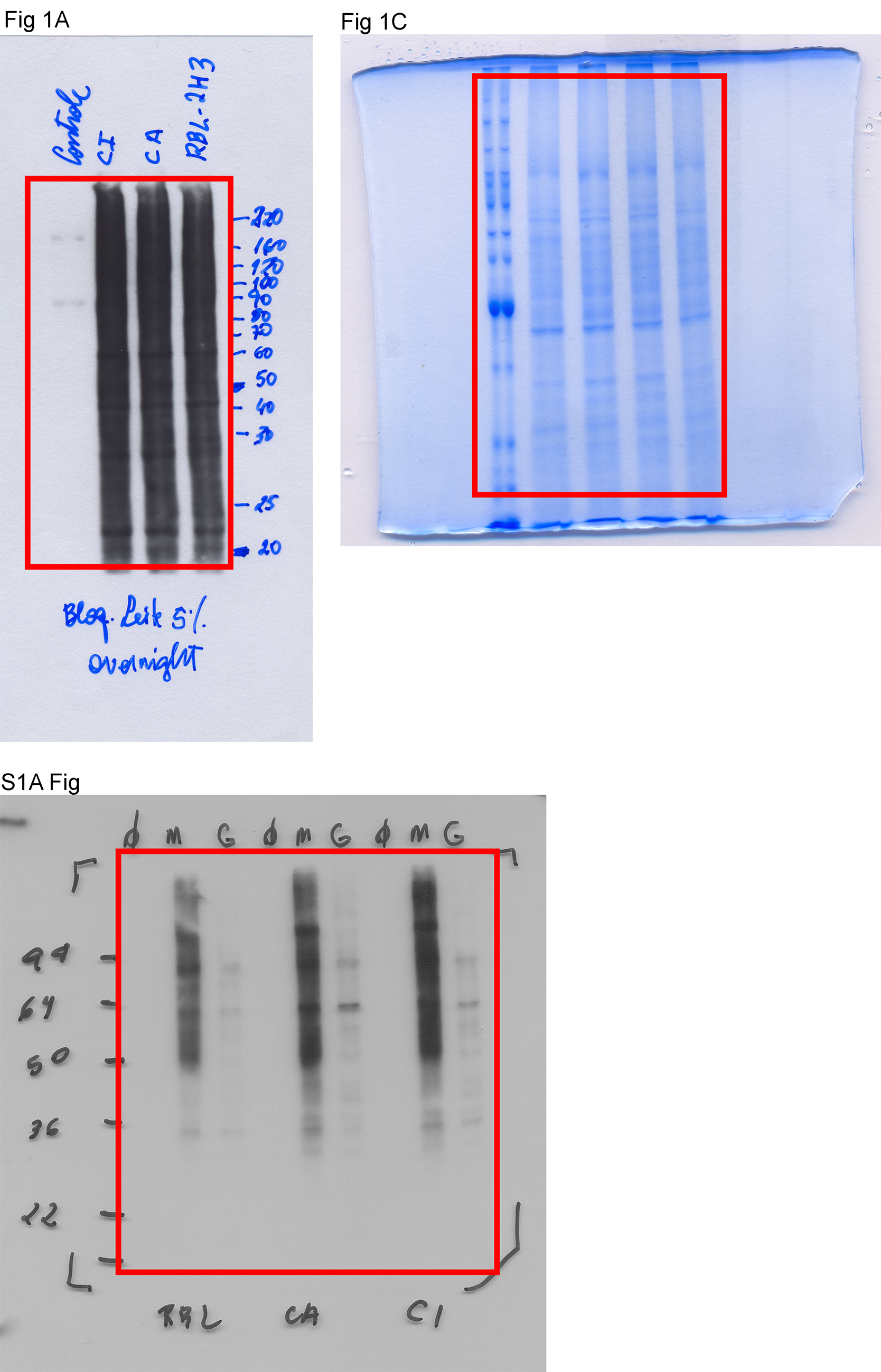

Supplement: S4 Fig — Original film from Fig 1A was inverted to follow the same pattern as electrophoresis gel of same samples and facilitate visualization. Uncropped picture of gel from Fig 1C. Original film from S1A Fig. (TIF) [file pone.0139888.s004.tif]
